# Agenesis of the Left Pulmonary Artery Associated With Hypoplasia of the Homolateral Lung: An Unusual Cause of Recurrent Respiratory Infection

**DOI:** 10.7759/cureus.70811

**Published:** 2024-10-04

**Authors:** Mohammed Musallam, Ibtissam El Ghazouani, Afaf Thouil, Hatim Kouismi, Cherifa Gounane

**Affiliations:** 1 Department of Pneumology, Centre Hospitalier Universitaire Mohammed VI Oujda, Oujda, MAR; 2 Faculty of Medicine and Pharmacy, Université Mohammed Premier Oujda, Oujda, MAR; 3 Department of Respiratory Diseases, Research and Medical Sciences Laboratory, Hôpital Universitaire International Mohammed VI, Oujda, MAR; 4 Department of Respiratory and Allergic Diseases, Hôpital Universitaire International Mohammed VI, Oujda, MAR; 5 Department of Pneumology, Centre Hospitalier d'Avignon - Hôpital Henri Duffaut, Avignon, FRA

**Keywords:** left pulmonary artery agenesis, pulmonary hypertension, pulmonary hypoplasia, rare congenital anomaly, respiratory tract

## Abstract

Agenesis of the left pulmonary artery represents a rare malformation, accounting for a small fraction of all cases of congenital heart disease. It is characterized by the absence of the left pulmonary artery, which can lead to respiratory and cardiac complications. This anomaly can appear on a standard chest X-ray, but it must be confirmed by a chest CT scanner, which makes it possible to visualize the absence of the left pulmonary artery and the associated parenchymal anomalies. The involvement of these patients depends on the evolution of their clinical condition, which may require symptomatic treatment and surgical intervention.

We present a case of agenesis of the left pulmonary artery in conjunction with hypoplasia of the corresponding lung, diagnosed in a 45-year-old patient following a recurrent respiratory infection.

## Introduction

Agenesis of the pulmonary artery is a rare congenital vascular anomaly that Fraentzel first documented in 1868 [1]. This vascular anomaly is rare and affects about 1% of congenital heart disease. The condition primarily affects the right pulmonary artery [2]. Agenesis of the left pulmonary artery is defined as the complete absence of the left pulmonary artery, which results in hyperplasia of the left lung and is associated with a range of respiratory and cardiac complications [3].

This anomaly can result in significant morbidity and mortality and should be considered in the presence of symptoms such as recurrent respiratory infections, dry cough, or exertional dyspnea. A CT chest scanner with contrast is a diagnostic imaging technique employed to visualize pulmonary artery agenesis and associated anomalies. The severity of the symptoms determines the need for management, which primarily involves a conservative approach. This study presents a case of agenesis of the left pulmonary artery associated with hypoplasia of the homolateral lung.

## Case presentation

This case report details the clinical presentation of a 45-year-old patient with a history of recurrent respiratory infections and first-degree consanguinity. There was no evidence of tuberculosis contagion or exposure to tobacco. The patient's medical history reveals a diagnosis of respiratory illness at the age of 25, marked by the onset of morning bronchorrhea. This was accompanied by stage III mmRc exertional dyspnea, without cyanosis or hemoptysis. The patient's condition subsequently evolved in the context of intermittent fever and the preservation of their general condition. He had previously been treated as an outpatient on several occasions. One week prior to his scheduled consultation, his dyspnea had progressed to stage IV mmRc, which is associated with orthopnea and an increased volume in both lower limbs.

**Figure 1:**
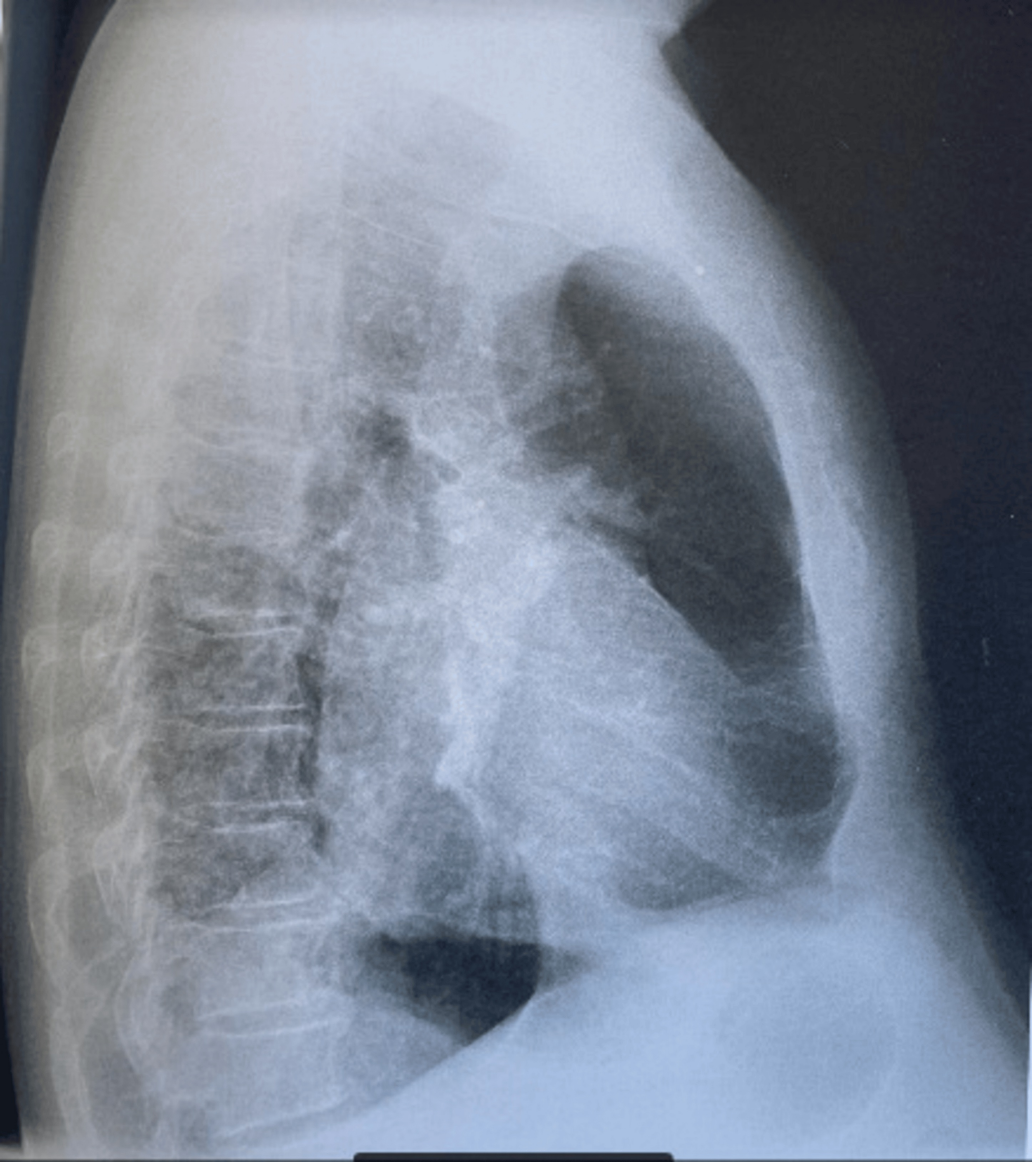
Lateral chest X-ray Left basal lung opacity that obliterates the edge of the heart, associated with atelectasis and a small left lung field

A fever with a temperature of 39 °C, a heart rate of 120 beats per minute, a respiratory rate of 28 cycles per minute, and a saturation of 94% on room air was discovered during a clinical examination. Additionally, nail clubbing was observed. Pulmonary auscultation revealed left basithoracic bronchial rales, and cardiac auscultation revealed a systolic murmur at the tricuspid valve focus. A chest x-ray (Figure 1.) was performed and showed a left basal lung opacity obliterating the edge of the heart with left mediastinal attraction related to atelectasis associated with a small left lung field appearance.

**Figure 2:**
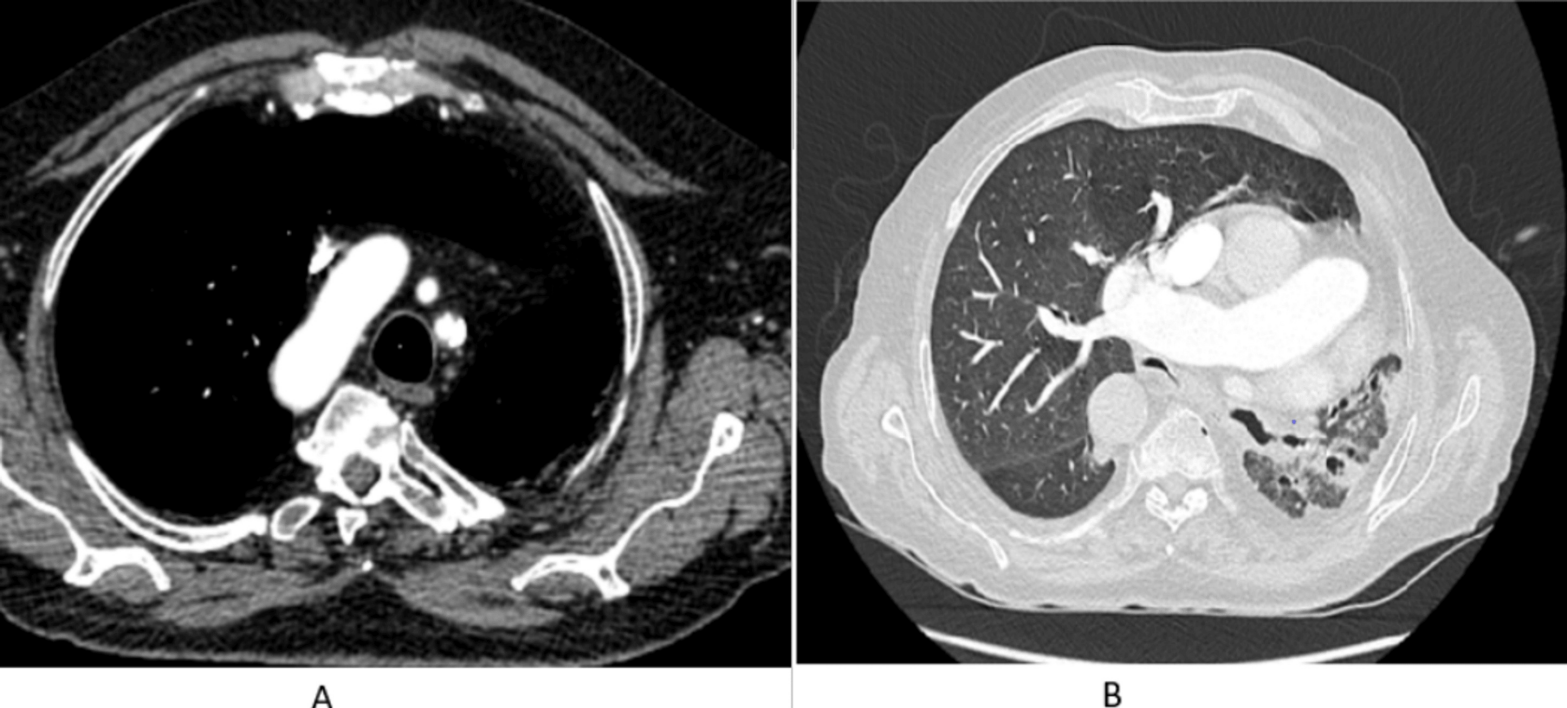
(A) Mediastinal window of the thoracic angioscan demonstrating the absence of the left pulmonary artery in conjunction with a prominent large-vessel malformation involving the right thoracic aorta. (B) Chest CT parenchymal window demonstrating hypoplasia of the left lung and left inferior lobar varicose bronchiectasis with an infectious appearance CT: computed tomography

A complementary CT scan with injection was performed (Figure 2) and showed the absence of individualization of the left pulmonary artery with hypoplasia of the homolateral lung. Conversely, there was significant left-systemic collaterality via the intercostal, bronchial, internal thoracic, and diaphragmatic arteries, as well as large-vessel malformation with a right-sided thoracic aorta and infectious LIG varicose bronchiectasis. The results of the bacteriological testing (quantifierons and Koch's bacillus test in sputum) were negative.

**Figure 3:**
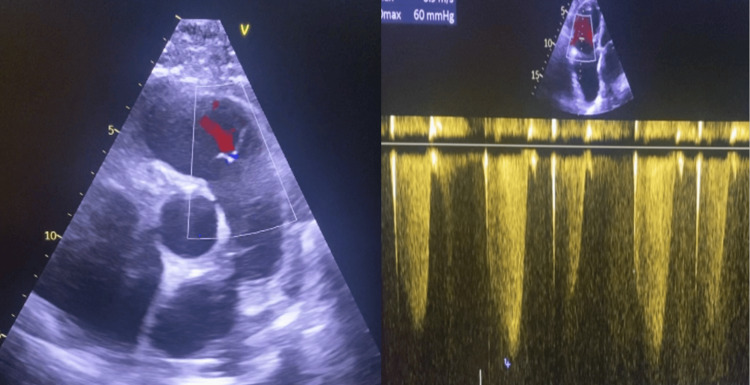
ETT in two-dimensional mode showing an apical section of four cavities centered on the right cavity It reveals the absence of the left pulmonary artery and notable dilation of the right atrium and right ventricle. The inferior vena cava velocity is 3.9 meters per second, and the right ventricle and right atrium gradient are 60 millimeters of mercury. ETT: echocardiogram

There are no associated cardiovascular malformations on ultrasound (Figure 3); in particular, there is no ventricular septal defect or patent ductus arteriosus. On the other hand, we found significant right atrial and right ventricular dilatation with tricuspid regurgitation with a velocity of 3.9 m/s and a right ventricle-to-right atrium gradient of 60 mmHg, consistent with severe pulmonary arterial hypertension (PAH).

Spirometry revealed a mixed obstructive and restrictive syndrome (FEV1: 2.60 L, or 65% of the predicted value; FVC: 3 L, or 65%; TLC: 60% or 3.60 L). Considering the radio-clinical results described previously, the diagnostic of primitive homolateral pulmonary hypoplasia in association with agenesis of the left pulmonary artery complicated by severe PAH and the Table of Global Heart Failure were maintained.

A surgical cure was proposed to the patient, who declined. Instead, he was placed on a conservative treatment plan, which included antibiotic therapy, respiratory physiotherapy, and rigorous clinical, radiological, and cardiographic monitoring.

## Discussion

Unilateral primary pulmonary artery agenesis is a rare congenital malformation. The condition was initially described by Fraentzel in 1868; however, it was not until 1952 that it was visualized on angiography by Madoff et al. [1]. The incidence of this condition is approximately 1 in 200,000, representing only 1% of all cases of congenital heart disease [2,3].

As stated by Sherrick et al. [4], these structures are more frequently observed on the right than on the left. Furthermore, 58% of cases of right pulmonary artery agenesis are isolated, in comparison to only 19% of cases of left pulmonary artery agenesis.

The phenomenon of homolateral lung hypoplasia can be attributed to the parallelism between the processes of vascular development and alveolar growth. A pulmonary artery with stunted growth results in a peripheral alveolar growth defect, which in turn gives rise to diffuse harmonic pulmonary hypoplasia [5]. It is seldom symptomatic and is typically identified in childhood as a consequence of complications or as part of a malformative cardiovascular syndrome. The most common associated cardiovascular malformations are tetralogy of Fallot, ventricular septal defect, right aortic arch, transposition of the great vessels, and abnormal venous return or persistent ductus arteriosus. It is crucial to identify these associated malformations following diagnosis, as they significantly impact the prognosis and mortality of the disease [6].

Dyspnea and fatigability have been frequently documented in the literature, as well as in our patient. These symptoms appear to be more specific to the malformation, as they resolve after pneumectomy [7].

A potential complication of this condition is hemoptysis, which occurs in 20% of cases. Another possible consequence is PAH, which develops in 25% of cases due to hypertrophy of the systemic vasculature. Additionally, exertional dyspnea may occur in 40% of cases, recurrent respiratory infections in 37%, asthma in 75%, and acute cardiogenic pulmonary edema [8].

In this case, the patient presented with a history of recurrent respiratory infections, exertional dyspnea, and severe PAH. A variety of signs identified through standard radiological techniques can be employed to elicit a diagnosis. These include asymmetry of vascularization, absence of a hilar shadow, a small hyper-clear lung with mediastinal attraction, and, in some cases, compensatory emphysema of the contralateral lung [9].

A diagnosis is typically established through the use of angiography. Nevertheless, the thoracic angioscanner is currently demonstrating itself to be an efficacious and minimally invasive instrument. It is capable of promptly eliminating pulmonary embolism, the primary differential diagnosis in adults, from consideration. Furthermore, it allows for the analysis of the pulmonary veins, the elimination of Felson's venolobar syndrome, and the assessment of the pulmonary parenchyma, including the possibility of complete pulmonary agenesis and hypoplastic lung with or without ventilation. Bronchoscopy is a valuable tool for visualizing a rudimentary bronchus. Nevertheless, MRI is not an effective diagnostic tool.

In the context of scintigraphy, the absence of perfusion in a given lung field is observed, with the persistence of normal or only slightly diminished ventilation and the absence of gas retention during the evacuation of Xenon 133 [10]. At this juncture, two discrete diagnoses remain: pulmonary artery thrombosis or stenosis.

A follow-up cardiac ultrasound is often useful for detecting malformations, as it can be employed to ascertain whether PAH has developed. It is not uncommon for respiratory function tests to yield normal results. However, they may also indicate a reduction in lung capacity and expiratory flow, in addition to an increase in airway resistance. This is attributable to a reduction in pulmonary vascularization, which is compensated for by the development of bronchial and collateral arteries [11]. The efficacy of the physical stress test is contingent upon the age of the subject and the presence of clinical symptoms.

The prognosis is contingent upon the presence of associated malformations, the site of agenesis (right or left), and the date of onset of clinical signs. It is notable that a considerable number of individuals with this condition still succumb to their illness before reaching the age of 20. The survival rate for patients with right-sided agenesis is 10%, compared with 45% for those with left-sided pulmonary agenesis. It is postulated that this more severe prognosis is attributable to the greater mediastinal displacement that occurs in cases of right lung agenesis. The placement of an expandable prosthesis in the empty hemithorax has been demonstrated to correct this mediastinal deviation [12].

The scope of management is frequently constrained to clinical monitoring, respiratory function with stress testing, and cardiac ultrasound, with no consensus on the optimal frequency of these procedures. The approach must be tailored to the specific complexities of each case. It is recommended that asymptomatic forms be left untreated. Only symptomatic patients can benefit from medical treatment with vasodilators in the case of pulmonary hypertension and embolization in the case of hemoptysis. In the event of an unsuccessful endovascular treatment of hemoptysis or the case of repeated infections, surgical intervention in the form of a pneumonectomy is imperative.

Our patient was offered surgery, which he refused, so he was treated conservatively.

## Conclusions

Agenesis of the left pulmonary artery with pulmonary hypoplasia is a rare malformation. It may persist for a long time without symptoms or manifest with complications such as recurrent respiratory infections, cough, exertional dyspnea, and hemoptysis. An early diagnosis is crucial, as the prognosis can be fatal. Cross-sectional imaging based on an angioscanner is necessary to allow for simultaneous analysis of vessel opacification and lung parenchyma. The treatment plan is typically based on conservative methods.
